# Exercise Interventions for Improving Flexibility in People with Multiple Sclerosis: A Systematic Review and Meta-Analysis

**DOI:** 10.3390/medicina55110726

**Published:** 2019-11-02

**Authors:** Marta Torres-Pareja, Miguel A. Sánchez-Lastra, Laura Iglesias, David Suárez-Iglesias, Nuria Mendoza, Carlos Ayán

**Affiliations:** 1 Research Group DEPORSALUD (Physical Activity and Health, Studies in Sports Performance, Disability and Gender), Faculty of Sports Sciences, University of Castilla La-Mancha, A Carlos III s/n, E-45071 Toledo, Spain; 2 HealthyFit Research Group, Department of Special Didactics, Faculty of Educational Sciences and Sports, University of Vigo, Campus A Xunqueira s/n, E-36005 Pontevedra, Spain; misanchez@uvigo.es; 3 Faculty of Education and Sport Science, University of Vigo, E-36005 Pontevedra, Spain; lauraiglesiasco@gmail.com; 4 VALFIS Research Group, Institute of Biomedicine (IBIOMED), Faculty of Physical Activity and Sports Sciences, University of León, 24071 León, Spain; dsuai@unileon.es; 5 Facultad de Ciencias de la Salud, Universidad Francisco de Vitoria. Ctra Pozuelo a Majadahonda 11-515 KM 1.800, 28223 Pozuelo de Alarcón, Madrid, Spain; nuria.mendoza@ufv.es; 6 Well-Move Research Group, Department of Special Didactics, Faculty of Educational Sciences and Sports, University of Vigo, Campus A Xunqueira s/n, E-36005 Pontevedra, Spain; cayan@uvigo.es

**Keywords:** flexibility, multiple sclerosis, range of motion, stretching

## Abstract

*Background and objectives:* People with multiple sclerosis (MS) often experience limitations in joint range of motion, which is linked to spasticity and continued inactivity. Low flexibility levels in this population have been linked to postural problems and muscular pain. Therefore, the purpose of this study was to conduct a systematic review and a meta-analysis aimed at identifying the characteristics and methodological quality of investigations studying the effects of exercise interventions on the flexibility levels of people with MS. *Materials and Methods:* Three electronic databases (MEDLINE/PubMed, SPORTDiscus and Scopus) were systematically searched up to May 2019 for intervention studies focused on the effects of exercise on the flexibility levels of people with MS. A meta-analysis, including randomized controlled trials (RCT), which reported information regarding the effects of exercise on flexibility, was also conducted. The methodological quality of included studies was assessed using the Physiotherapy Evidence Database, and the Quality Assessment Tool for Before–After Studies, with no control group. The quality of the information reported, regarding the programs conducted, was assessed by means of the Consensus on Exercise Reporting Template (CERT) scale. *Results:* Seven studies, four RCTs and three uncontrolled investigations were finally selected. The methodological quality of the RCTs was considered “poor” in one study, and “good” and “excellent” in two studies and one investigation, respectively. The three uncontrolled studies showed a methodological quality between “fair” and “poor”. Following the CERT scale, four studies were graded as “high” and three as “low”. Findings from the meta-analysis indicated no significant effects on hamstring flexibility, or the range of motion in the hips, knees or ankles. *Conclusions:* There is preliminary evidence from individual studies which indicates that people with MS can improve their lower limb flexibility following participation in physical exercise programs, but the meta-analysis did not confirm these findings.

## 1. Introduction

Multiple sclerosis (MS) is a complex condition, characterized by the inflammation of the central nervous system, and it causes axonal or neuronal loss, demyelination and astrocytic gliosis [1]. It is one of the most common neurological disorders worldwide; an estimated 2.3 million people live with MS [2]. In many countries, it is the main cause of non-traumatic neurological disability in young adults [2]. Its incidence is higher among women, and in the Northern Hemisphere [3]. The course of the disability cannot be predicted, and its clinical presentation may vary greatly from patient to patient. There is no known cure for MS [4]. Current management guidelines are focused on reducing its exacerbation, through therapies aimed at reducing symptoms [4]. In the last 25 years, these therapies have shown to have a positive effect on these parameters [5], but the individual with MS still experiences an enormous impact on their level of function, which has a considerable negative effect on overall quality of life.

Physical exercise has been postulated as one of the non-pharmacological strategies of interest, due to its low cost and positive effects on the physical and mental health of the MS population [6]. In general, the majority of research on the effects different exercise types have on this group focuses on training programs, which aim to improve cardiovascular and muscular fitness, in view of the effects that these generally have on two symptoms of great prevalence, namely, fatigue and muscular weakness [7]. Most of the previous studies on this subject have focused on analyzing the effects of aerobic and resistance training, hoping to identify basic guidelines for the prescription of physical exercise for people with MS.

In this regard, it should be mentioned that a training pyramid has been proposed, with the purpose of prescribing physical exercise for persons with MS. The basis of this pyramid is formed of the passive range of motion (ROM) exercises, and then it progresses towards active flexibility exercises [8]. This is because persons with MS often experience limitations in articular movement, which are linked to spasticity and continued inactivity [9]. Reduced levels of muscular flexibility have also been linked to postural problems and muscular pain in this population [10]. Nevertheless, even though flexibility is considered one of the basic components of a healthy physical condition, no systematic reviews contributing to the information on the effects of exercise programs on the flexibility of persons with MS have been published so far, or basic guidelines for prescribing these programs. Therefore, there is a need to conduct a systematic review to provide scientific evidence regarding the type of programs specialists in neurorehabilitation should prescribe to persons with MS, as well as to describe the expected benefits of these programs. Under these circumstances, the objective of this study was to conduct a systematic review and a meta-analysis, aimed at identifying the characteristics and methodological quality of investigations that have studied the effects of exercise intervention on the flexibility levels of people with MS.

## 2. Materials and Methods

### 2.1. Search Process

The search strategy was selected and designed in order to find research studies which gave information on the effects of physical exercise on the flexibility of persons with MS. Three electronic databases were used for the search (*Medline/PubMed, SPORTDiscus and Scopus),* up to May 2019, using the key words and Boolean operators “Multiple Sclerosis” AND “Range of Motion” OR “Flexibility” OR “Stretching”.

### 2.2. Selection Procedure and Eligibility Criteria

Eligibility was assessed by one author and supervised by a second author. Those studies which, having proposed a physical exercise program for persons with MS, included variables relating to flexibility amongst their outcomes, were considered eligible. Investigations were excluded if: (a) the sample included participants with MS and other conditions, and data for each population was not reported separately; (b) exercise was included as an additional treatment arm, or it was performed as part of a combined therapy program and its effects could not be isolated; (c) the intervention was based on the performance of a single exercise training session; (d) the research was not published in a peer-reviewed journal written in English, French, Portuguese or Spanish.

The studies were examined independently, by reviewing the information which appeared in the titles and abstracts and classifying them as “selected” or “eliminated”. The studies which did not provide sufficient information were registered as “doubtful”, pending a subsequent reading of the full text.

Once this first selection was complete, the information was contrasted, and a second review was carried out with the assistance of the other co-authors. The studies which were then rejected were those which included: (a) combined exercise interventions which did not include a measure of flexibility; (b) interventions which included pharmacological treatment together with exercise. Finally, seven articles were chosen which fulfilled the requirements and which were related to the object of study of this review.

### 2.3. Data Extraction

The information in each study regarding design type, characteristics of the sample, the exercise program to be carried out, flexibility assessment tools, and effects of the program on flexibility, was extracted by a researcher onto a data log grid. The information was subsequently independently revised by a second researcher.

### 2.4. Assessment of Methodological Quality

The methodological quality of the studies that were considered randomized controlled trials (RCTs) was extracted directly from Physiotherapy Evidence Database (PEDro) [11]. If a study was not registered in this database, the PEDro scale was applied by two authors independently. The suggested cut-off points to categorize studies by quality were as follows: excellent (9–10), good (6–8), fair (4–5), and poor (<3) [12].

For the studies with no control groups, the “Quality Assessment Tool for Before–After Studies with No Control Group” [13] tool was applied by two authors independently. This tool includes 12 questions, and authors must define the quality of each study (“poor”, “fair” or “good”) according to how much risk of bias they consider exists. In either procedure, in cases of disagreement, advice was sought from a third author.

The quality of the information reported with regard to the characteristics of the programs conducted was assessed by means of the Consensus on Exercise Reporting Template (CERT) scale, applied by a single author [14]. The scale contains 16 questions with scores between 1 and 19. A score of <9 is considered “low” methodological quality, and a score of ≥9 is considered “high”.

### 2.5. Data Analysis

A meta-analysis, including RCTs which reported information regarding the effects of exercise on flexibility, both before and after the intervention, was performed when the same outcomes had been assessed in at least two studies in a comparable manner [15]. Pre-and post-intervention data was presented for the control and intervention groups as mean ± standard deviation (SD). In order to do this, standardized mean differences (SMD) and their 95% confidence intervals (CIs) were calculated, to assess the change in each selected variable. The SMD is the mean divided by the SD, and its calculation incorporated control and intervention sample sizes, pre- and post-intervention means, and SDs, for each of the selected outcome measures. To obtain the pooled effects, a fixed-effects model was used. In the event of a heterogeneity level (I^2^) over 30%, a random-effects model was also applied [16]. This same procedure was conducted in order to analyze the pooled effects of the programs in the intervention groups, taking into account only the data from these groups. Forest plots displaying SMD and 95% CIs were used to compare these effects in the pre- and post-intervention measurements in the intervention groups. SMDs were significant when their 95% CIs excluded zero, while pooled SMD values of less than ±0.2, ranging from ±0.2 to ±0.8, and greater than ±0.8 indicated the existence of small, medium, or large effects, respectively. In all the studies, the relative effect size (ES), by means of Cohen’s *d*, was calculated for all flexibility outcomes, by analyzing the intra-group pre- and post-intervention measurements. In addition, the absolute ES, comparing the groups in the study, was also calculated. These calculations incorporated the post-intervention sample sizes, as well as pre- and post-intervention means and SDs for each of the selected outcome measures. Following Cohen’s classification [17], ESs were divided into trivial (*d* ≤ 0.2), small (*d* > 0.2), moderate (*d* > 0.5), large (*d* > 0.8), and very large (*d* > 1.3). If the studies intended to be included did not report the required data, the authors were contacted and it was requested. All statistical analyses were performed using Stata 13.

## 3. Results

### 3.1. Designs and Samples

**Figure 1:**
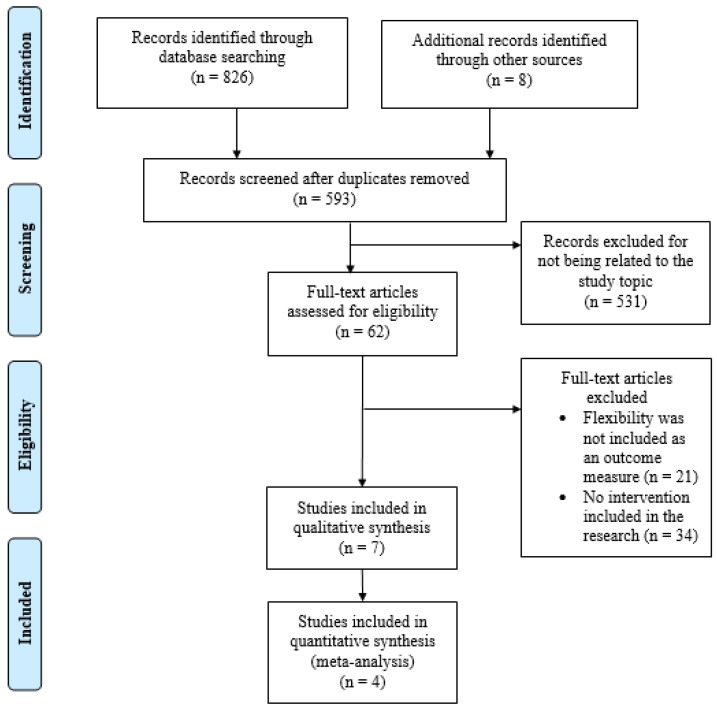
Flow chart of the systematic review process.

Of the 834 references located, 62 were initially selected. Following a reading of the full text, a total of seven studies (four RCTs [18,19,20,21] and three uncontrolled investigations [22,23,24]) were included in the final analysis (Figure 1). In accordance with the studies, which included information regarding the characteristics of the sample, a total of 163 participants (64.4% women), with an average age of 49.5 ± 5.5 years participated in the proposed interventions. The general characteristics of the studies included are shown in Table 1.

### 3.2. Quality Assessment

The methodological quality of the RCTs was considered “excellent” [18] and “good” [19,20] in three of the studies analyzed, and “poor” in one [21] (Table 2).

The three uncontrolled studies showed a methodological quality between “fair” [22,23] and “poor” [24] (Table 3).

Following the administration of the CERT scale, four studies were graded as “high” [18,19,20,22] and three [21,23,24] as “low” (Table 4).

### 3.3. Interventions

The selected studies carried out different interventions, based on the performance of aerobic exercise [23], strength exercises [21], or a combination of multiple forms of exercise (flexibility, balance and strength [18,22] or aerobic and strength [20]). Other research proposed interventions based on Pilates [19,21] or Tai chi [24]. Only one study included a specific flexibility program [21] (Table 1). The average duration of the training programs was 23.1 ± 7.1 weeks, with an average of 2 ± 0.8 sessions per week, with the exception of one study, which does not give the session times, but the times of the sets carried out; the average duration of a set was 67.5 ± 27.2 min.

### 3.4. Effects of the Programs on Flexibility

Of a total of seven studies, three provided information on the effects of the program on an intergroup and intragroup level [18,19,20]. The remaining four only provided information on an intragroup level [21,22,23,24]. Significant changes in different variables were observed after the completion of the proposed programs in five of the seven studies analyzed (Table 1). The ES of the different studies is presented in Table 5. The studies from Ponzano et al., (2017) [21], Pereira et al., (2012) [22] and Husted et al., (1999) [24] did not report sufficient data to calculate the ES.

#### 3.4.1. Lower Limb Range of Motion.

**Figure 2:**
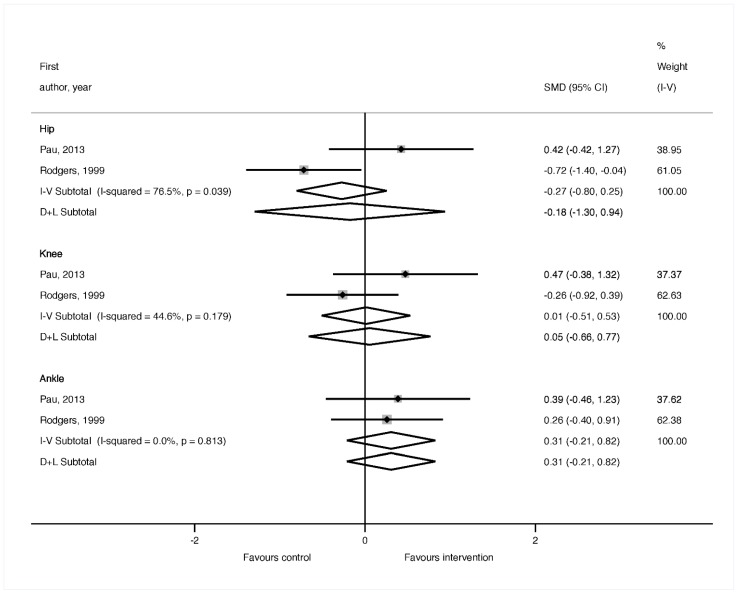
Forest plot of the meta-analysis comparing baseline and post-intervention effects in the intervention groups for the range of motion of the hip, knee and ankle during gait.

A total of three investigations [20,22,23] provided information on the effects of the exercise interventions on the lower limb ROM of the participants. All of these reported significant positive effects after the exercise program, which manifested in a static ROM improvement in the ankle plantar-flexion (right), knee flexion (right–left), hip flexion (right–left) and hip abduction (left) [22], hip flexion with knee extended (moderate to large ES), hip abduction (moderate to large ES), hip adduction (large to very large ES) and hip external rotation (very large ES) [23]. Improvements were also found in the ROM during the gait in the hip flexion–extension (small to moderate ES), knee flexion–extension (small to moderate ES), ankle dorsi–plantar–flexion (small to moderate ES) [20], ankle dorsi-flexion (large to very large ES), ankle plantar-flexion (large to very large ES), ankle angle at contact (large to very large ES), ankle angle at toe-off (moderate to large ES), knee flexo-extension (trivial ES), hip extension (trivial ES), hip flexo-extension (trivial ES), hip adduction (large to very large ES), hip abduction (very large ES), hip angle at contact adduction–abduction (very large ES) variables. When the meta-analysis was performed for the ROM, no significant effects were found comparing the baseline and post-intervention results in the intervention groups (Figure 2).

#### 3.4.2. Flexibility of the Posterior Kinetic Chain

**Figure 3:**
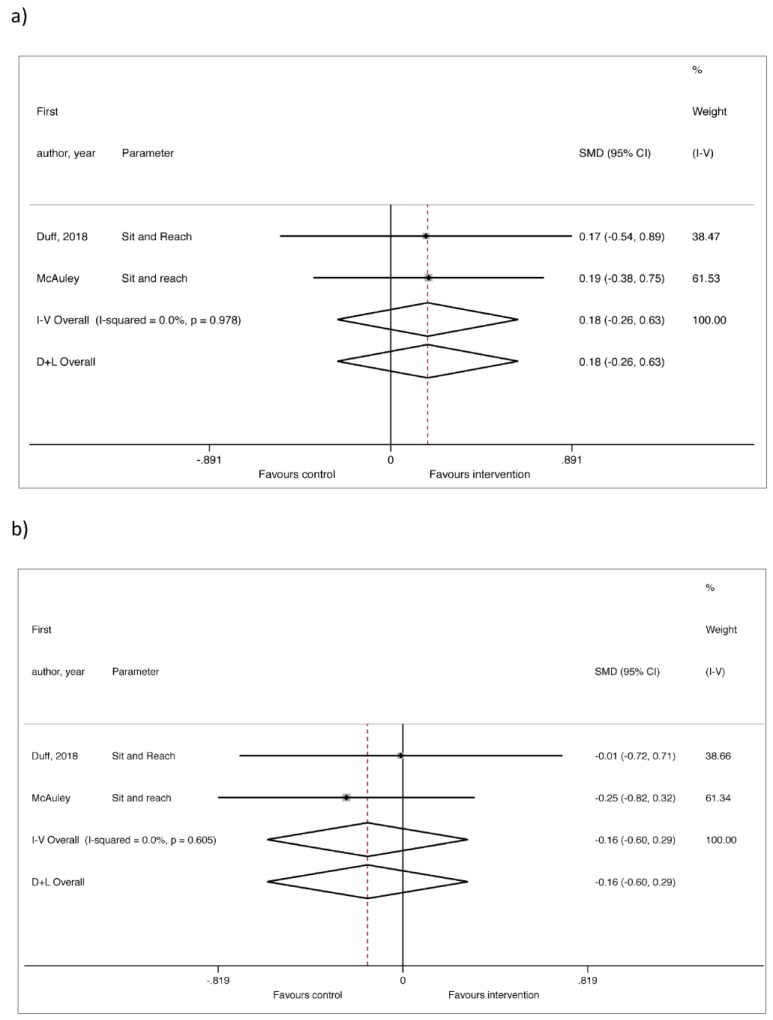
Forest plot of the meta-analysis for the Sit and Reach test only in the intervention groups (**a**) and compared to the controls (**b**).

Four of the articles reviewed measured the posterior kinetic chain [18,19,21,24]. There appear to be no significant differences in two of these [18,19]. However, in the study by Ponzano et al. [21], the posterior kinetic chain was assessed by means of the Sit and Reach test, and significant differences were found in two of the groups (G_1_ y G_3_; small to moderate ES). Significant differences were also found in G_1_ using the spinal mouse test, which assesses the morphology of the rachis on the sagittal plane (moderate to large ES). Finally, the study by Husted et al. [24] mentions an improvement of 28% between pre- and post-test results, using the Hamstring Flexibility Test to measure the flexibility of the hamstrings through the spinal column. When the meta-analysis was performed for the Sit and Reach test, no significant effects were found, either when comparing the post-intervention effects in the intervention groups (Figure 3a, *n* = 39), or when comparing the intervention to the control groups (Figure 3b, *n* = 78).

#### 3.4.3. Upper Limb Range of Motion

The upper limb ROM (shoulder, elbow and wrist), differentiating the right side from the left side, was assessed in just one of the articles reviewed [22]. The authors reported that the intervention had significant effects on the extension (right and left) and abduction (left) of the shoulder, on the flexion (right and left) of the elbow and on the extension (right) of the wrist.

#### 3.4.4. Upper Limb Flexibility

Measurements of upper limb flexibility were taken only by means of the Back Scratch test in one of the studies [18], in which no significant differences were found between the pre-test and post-test results, when adjusted by age. However, when adjusted by gender and by pre-intervention values, improvements were found in both groups.

## 4. Discussion

In this research, scientific evidence of the effectiveness of physical exercise, for the improvement of aspects relating to flexibility in persons with MS, has been examined. In order to achieve this objective with maximum precision, the search was programmed to find the highest number of studies which focused only on exercise interventions, although it was not limited to the study of RCTs, for various methodological reasons. Firstly, when a limited number of RCTs are found, it is difficult to draw solid conclusions. Therefore, the inclusion of non-RCT studies may be useful to obtain a better view of the current interventions, with a view to informing future research [25]. Secondly, non-RCT studies may provide relevant information on feasibility, adverse effects, or response rate, in said interventions [26]. Finally, non-RCT studies may include important detailed information on the characteristics of the interventions carried out, such as the number and duration of sessions, type of exercises, rests, intensity, volume, etc. Thus, in this review, the spectrum of results is wider, providing greater clarity as to the state of the problem, having extracted the data and conclusions.

An important finding in this research is the fact that only three of the seven studies selected demonstrated good methodological quality. This would suggest that current scientific evidence is limited and, therefore, future RTCs are necessary in this line of investigation. However, valid information can be extracted from all the studies analyzed, since they provided interesting data regarding the characteristics of the interventions performed, which could be useful for rehabilitation professionals who deal with this population.

The results obtained in the studies reviewed suggest that, when the intention is to increase ROM in persons with MS, aerobic and strength exercises appear to be the most effective. On the other hand, when the intention is to increase muscular extensibility, alternative physical therapies, such as Pilates and Tai chi, would appear to be the most effective strategies. There is a remarkable lack of studies which base their research on exercises that seek only to improve flexibility, or which propose a single stretching program to see the resulting effects. This may be because exercise-based rehabilitation therapies focus more on strategies designed to reduce the impact of principal symptoms, such as fatigue (through aerobic training) [27] or muscular weakness [28]. Stretching programs have generally aimed to treat of spasticity [29], and so the effect of this therapy on the ROM or muscular extensibility of persons with MS who are not affected by this symptom remains unknown.

Despite the limited number of studies found, it was possible to conduct a meta-analysis in which the data could be synthesized. Meta-analysis allows us to evaluate whether the size of the effect is consistent and if the effect may be considered strong, and, therefore, the size of the effect may be estimated with greater precision than with a single study. Moreover, if the size of the effect varies, that variation may be described and, potentially, explained [30].

The results of the conducted meta-analysis indicated that there were no significant improvements in any of the variables analyzed. In the case of the Sit and Reach test, although included studies showed a great degree of homogeneity, these results could be influenced by the type of test chosen to assess flexibility. A tendency towards improvement in favor of the intervention group could be observed only when the evolution of the control group is not considered. With regard to ROM assessment of hip, knee and ankle joints, significant effects, in favor of the intervention groups, were not shown. The only joint in which a tendency towards improvement was observed in was the ankle. It would be worth considering whether the effects of exercise tend to occur sooner in the ankle due to its relative anatomic simplicity, compared to the knee or the hip, which involve greater muscle groups, and where factors such as propioception and balance play a fundamental role [31]. The limited sample size, and the heterogeneity of the studies included, limits the possibility of explaining the results, and, therefore, studies with greater homogeneity in their measurement protocols are necessary, in order for more decisive conclusions to be reached.

Systematic reviews serve to provide more certainty about the scientific evidence presented in a research paper. The results of these reviews form the basis for the development of new research on the same topic. In this regard, the present review brings to light that the majority of studies related to the effects of exercise on the flexibility of persons with MS focused on the lower limbs, perhaps due to these areas being more affected than the upper limbs, in persons with MS [32]. However, mobility in the upper body is fundamental, as three out of four patients with MS face a functional decrease in the upper extremity [33], with a negative impact on daily activities, such as grooming, showering, eating and writing, which reduces quality of life and results in greater dependency [34]. Therefore, future lines of research should analyze the effects of exercise programs on upper body ROM.

As far as the authors are aware, this is the first review study which focuses on the effects that exercise programs have on flexibility in persons with MS. However, despite its originality, this study presents a series of limitations which should be recognized. First, a small number of studies were found, and their methodological quality left room for improvement. Second, the samples were composed of persons affected on a low level (EDSS 1-4), and the majority of the studies were focused on the lower limbs. Third, the low number of RCTs required that non-RCT studies were also included in the review. Fourth, the heterogeneity of exercise interventions and the outcomes measured should also be considered. Therefore, scientific evidence regarding the effect of exercise programs on MS can be rated as weak and could be improved upon. Additionally, there are inherent limitations in the design of this study, mainly due to language restrictions and the fact that grey literature was not sourced, factors which would affect the number of studies ultimately located.

## 5. Conclusions

There is preliminary scientific evidence from individual studies, which would indicate that persons affected with MS can improve their lower limb flexibility following participation in physical exercise programs, but the meta-analysis did not confirm these findings. Future, quality studies are necessary to verify the results, and these should take into account the effect that this type of program has on the upper limbs, in order to be able to form solid prescriptions, intended to improve flexibility among persons with MS.

## Figures and Tables

**Table 1:** Characteristics of the studies included in this review.

First Author, Year (Design)	Participants	Interventions	Outcomes (Test)	Results
McAuley, 2015 [18] (RCT)	*n* = 48 (75% women) Score EDSS: <6.5 IG: *n* = 24 (75% women) Score EDSS: NR Age: 59.62 ± 1.43 years Disease duration: 18.10 ± 9.42 years MS type: RR: *n* = 16 (66.7%) SP: *n* = 3 (12.5%) PP: *n* = 1 (4.2%) NR: *n* = 4 (16.6%) CG: *n* = 24 (75% women) Score EDSS: NR Age: 59.78 ± 1.50 years Disease duration: 19.85 ± 9.42 years MS type: RR: *n* = 16 (66.7%) SP: *n* = 2 (8.3%) PP: *n* = 0 NR: *n* = 6 (25%)	Combined Exercises Program: “FlexToBa DVDs” (balance, strength and flexibility) Duration: 6 months Development: - The first two weeks of each month: Volume: 1 to 2 sets of 8 to 10 reps Intensity: Borg 6–20 RPE scale of 10–12 - The last two weeks of the month: Volume: 2 sets of 10 to 12 reps Intensity: Borg 6–20 RPE scale of 13–15 Volume: 6 progressive exercise sessions/Each session containing two sets of 11 to 12 different exercises Frequency: 3 times/week (non-consecutive days) Intensity: All exercises began with the lower resistance band and advanced to the thicker band	Flexibility - Upper body (Back Scratch test) - Lower body (Sit and Reach test)	- Sit and Reach test: Intragroup: IG: Pre: −0.45 cm ± 0.84/Post: 0.62 cm ± 0.95 Post-adjusted: 0.61 cm ± 0.57 CG: Pre: −0.48 cm ± 0.84/Post: 0.44 cm ±0.95 Post-adjusted: 0.27 cm ± 0.57 Intergroup: Not-significant values *p*-value pre: 0.80/*p*-value post: 0.68 *p*-value post- adjusted: 0.68 - Back Scratch test: Intragroup: IG: pre: −4.52 cm ± 1.30/Post: −4.19 cm ± 1.06 Post-adjusted: −4.50 cm ± 0.54 CG: Pre: −5.28 cm ± 1.30/Post: −6.29 cm ± 1.06 Post-adjusted: −6.02 cm ± 0.54 Intergroup: *p*-value pre: 0.38/*p*-value post: 0.16 *p*-value post-adjusted: 0.05 *
Duff, 2018 [19] (RCT)	*n* = 30 (77% women) Score EDSS: NR IG: *n* = 15 (80% women) Score EDSS: NR Age: 45.7 ± 9.4 years Disease duration: NR MS type: RR: *n* = 14 (93%) SP: *n* = 0 PP: *n* = 1 (7%) CG: *n* = 15 (73% women) Score EDSS: NR Age: 45.1 ± 7.4 years Disease duration: NR MS type: RR: *n* = 11 (73%) SP: *n* = 2 (13%) PP: *n* = 2 (13%)	“Pilates exercise program and physiotherapy massage” Duration: 12 weeks Development: - Exercises in the standing position on the CoreAlign apparatus and floor mat work - Each session started with a warm-up and ended with a cool-down IG: Pilates + massage Volume: 50 min—Pilates 60 min—massage Frequency: 2 times/week 1 time/week Intensity: Existing exercises progressed in difficulty, and new exercises were introduced over the study period based on each participant’s individual performance. CG: Massage Volume: 60 min—massage Frequency: 1 time/week	Flexibility - Lower body, posterior kinetic chain (Sit and Reach test)	- Sit and Reach test: Intragroup: IG: Pre: 23.4 cm (11.4)/Post: 25.4 cm (11.0) CG: Pre: 28.4 cm (10.8)/Post: 30.3 cm (9.5) Intergroup: IG: 2.0 cm (−1.8 to 5.7) CG:1.9 cm (−2.0 to 5.8) *p*-value = 0.98
Pau, 2017 [20] (RCT)	*n* = 22 (45% women) Score EDSS: NR IG: *n* = 11 (45% women) Score EDSS: 3.6 ± 0.9 Age: 47.4 ± 10.8 years Disease duration: NR MS type: RR: *n* = 11 (100%) CG: *n* = 11 (45% women) Score EDSS: 3.4 ± 1.1 Age: 44.5 ± 13.5 years Disease duration: NR MS type: RR: *n* = 11 (100%)	“Combined exercises program” (aerobic and strength training) Duration: 24 weeks Development: Total volume: 60 min Frequency: 1 time/week Warm-up: Cycle-ergometer and stretching exercises for upper and lower limbs and trunk muscles Volume: 10 min Intensity: 30% of the maximum workload previously calculated by means of a cardiopulmonary test (CPT) Main Part: (Aerobic training) Volume: 20 min Intensity: 50% of the maximum value calculated for each participant on the basis of his/her CPT and progressively increased every week up to 80% of maximum work rate (Strength training) Volume: 20 min/1 set of 8 reps to progress until 3 sets of 12 reps Intensity: 15% (1RM) to progress until a 30% (1RM). Rest: 2–3 min/sets Cool-down: relaxation, postural control, spine mobility exercises and post-stretching Volume: 10 min	Flexibility - Dynamic ROM of the hip, knee and ankle (goniometer)	- Dynamic ROM during the gait cycle: Hip flexion–extension: Intragroup: IG: Pre: 42.70º ± 9.61/Post: 47.04° ± 10.13 *p*-value = 0.029 * CG: Pre: 42.71° ± 6.00/Post: 43.54° ± 3.88 Knee flexion–extension: Intragroup: IG: Pre: 52.88° ± 9.60/Post: 57.71° ± 10.06 *p-*value: 0.047 * CG: Pre: 50.75° ± 14.46/Post: 51.91° ± 12.98 Ankle dorsi–plantar–flexion: Intragroup: IG: Pre: 23.60° ± 5.81/Post: 26.08° ± 6.53 *p*-value = 0.043 * CG: Pre: 25.06° ± 10.14/Post: 25.17° ± 8.17
Ponzano, 2017 [21] (RCT)	*n* = 22 (NR %women) Score EDSS: ≤6.5 IG_1_: *n* = 8 (NR %women) Score EDSS: 4 ± 2 Age: 50 ± 18 years Disease duration: NR MS type: RR: *n* = 8 (100%) IG_2_: *n* = 7 (NR %women) Score EDSS: 3 ± 2 Age: 52 ± 10 years Disease duration: NR MS type: RR: *n* = 7 (100%) IG_3_: *n* = 7 (NR %women) Score EDSS: 2 ± 2 Age: 45 ± 6 years Disease duration: NR MS type: RR: *n* = 7 (100%)	Three groups of training: 1. Flexibility training 2. Strength training 3. Pilates program Duration: 16 weeks Development: Each training session began with a warm-up including joint mobility and muscle flexibility exercises IG_1_: static stretching protocols Volume: 11 exercises/3 sets of 30 s Rest: 30 seg/sets Frequency: 2 times/week IG_2_: elastic bands Volume: 11 exercises/3 sets of 10 reps Rest: 30 seg/sets Frequency: 2 times/week IG_3_: Pilates protocol Volume: 12 exercises/2 sets of 8 reps Rest: 30 seg/sets Frequency: 2 times/week	The three groups were tested three times: - T0: after a month - T1: two months after T0 - T2: two months after T1 Flexibility: - Posterior kinetic chain (Sit and Reach test and Spinal Mouse test).	No significant variations concerning unlisted parameters emerged from this research. - Spinal Mouse test: IG_1_: T0 and T2 (*p* < 0.05, −55%, ES = 0.67) “Inclination line test between ThSp1 and S1” IG_2_ and IG_3_: NR - Sit and Reach test: IG_1_: T0 and T2 (*p* < 0.05, +15%, ES = 0.36) IG_2_: NR IG_3_: T0 and T2 (*p* < 0.05, +15%, ES = 0.4)
Pereira, 2012 [22] (Descriptive longitudinal quantitative)	*n* = 4 (100% women) Score EDSS: NR Age: 45.5 years (between 33–53 years) Disease duration: 7 years MS type: RR: *n* = 4 (100%)	“Combined exercises program” by physiotherapy (strength, flexibility and balance) Duration: 30 sets Development: Home session; exercises of strength, flexibility and balance Volume: 60 min Frequency: 1 time/week Type of exercise to improve ROM: - 10 joint mobilizations (shoulder, elbow, wrist, hip, knee and ankle) - 3 muscle stretching maintained for 30 s - The same pattern was followed on the contralateral side	Flexibility in three different measurements: - ROM of the ankle, knee, hip, shoulder, elbow and wrist during active movements performed in dorsal decubitus, ventral, sitting and orthodontic, respectively (goniometer)	- ROM (First, second, third measurement): Ankle dorsi-flexion: Right: 7.5°, 10°, 15° Left: 10°, 17.5°, 20° Ankle plantar-flexion: Right: 30°, 37.5°, 42.5° * Left: 40°, 42.5°, 45° Knee flexion: Right: 105°, 112.5°, 117.5° * Left: 92.5°, 112.5°, 122.5° * Hip flexion: Right: 25°, 40°, 57.5° * Left: 25°, 47.5°, 55° * Hip extension: Right: 17.5°, 20°, 25° Left: 20°, 17.5°, 20° Hip abduction: Right: 27.5°, 30°, 32.5° Left: 25°, 32.5°, 40° * Shoulder flexion: Right: 142.5°, 140°, 165° Left: 147.5°, 150°, 160° Shoulder extension: Right: 60°, 65°, 70° * Left: 52.5°, 62.5°, 70° * Shoulder abduction: Right: 142.5°, 150°, 170° * Left: 160°, 162.5°, 170° * Elbow flexion: Right: 127.5°, 132.5°, 135° * Left: 130°, 140°, 140° * Wrist flexion: Right: 80°, 82.5°, 87.5° Left: 70°, 75°, 82.5° Wrist extension: Right: 57.5°, 62.5°, 65° * Left: 67.5°, 67.5°, 70°
Rodgers, 1999 [23] (Non-randomized, non-controlled)	*n* = 18 (77.7% women) Score EDSS: 3.6 ± 2.1 Age: 43.2 ± 10.8 years Disease duration: NR MS type: NR	“Aerobic exercise program” Duration: 6 months Development: Cycle ergometry protocol (upright or recumbent ergometer depending on their ability level) Volume: 30 min Intensity: 65–70% age-predicted maximal heart rate Frequency: 3 times/week	Measurements of flexibility: - Range of motion during walking (ROM gait) - Passive Range of motion (PROM) “Using goniometers (°) and standard techniques (ankle, knee, and hip)”	- ROM during the gait cycle, mean (SD): Ankle dorsi-flexion: Pre: 7.1 (5.3)/Post: 77.0 (8.1)/*p-*value = 0.0007 * Ankle plantar-flexion: Pre: 101.0 (8.7)/Post: 108.9 (8.3)/*p-*value = 0.0001 * Ankle dorsi-plantar-flexion: Pre: 29.8 (7.1)/Post: 31.9 (8.8)/*p-*value = 0.204 Ankle angle at contact: Pre: 89.2 (5.3)/Post: 97.0 (7.3)/*p-*value = 0.0001 * Ankle angle at toe-off: Pre: 92.9 (9.1)/Post: 99.5 (10.7)/*p-*value = 0.003 * Knee flexion: Pre: 122.5 (9.3)/Post: 124.3 (11.0)/*p-*value = 0.178 Knee extension: Pre: 172.0 (6.4)/Post: 171.0 (6.1)/*p-*value = 0.339 Knee (flexo-extension): Pre: 49.5 (10.2)/Post: 46.6 (11.3)/*p-*value = 0.023 * Knee angle at contact: Pre: 165.1 (8.0)/Post: 165.6 (8.0)/*p-*value = 0.736 Knee angle at toe off: Pre: 142.4 (7.9)/Post: 143.4 (11.8)/*p-*value = 0.644 Hip extension: Pre: 176.0 (6.8)/Post: 172.0 (6.5)/*p-*value = 0.020 * Hip flexion: Pre: 151.1 (8.6)/Post: 151.0 (8.7)/*p-*value = 0.949 Hip flexo-extension: Pre: 24.9 (6.0)/Post: 21.0 (4.5)/*p-*value = 0.0029 * Hip angle at contact, flexo-extension: Pre: 156.6 (8.3)/Post: 157.8 (9.3)/*p-*value = 0.431 Hip angle at toe off, flexo-extension: Pre: 169.6 (6.9)/Post: 166.4 (7.3)/*p-*value = 0.052 Hip adduction: Pre: 189.3 (6.8)/Post: 196.7 (4.7)/*p-*value = 0.000 * Hip abduction: Pre: 172.2 (6.7)/182.2 (4.5)/*p*-value = 0.000 * Hip adduction-abduction: Pre: 16.5 (6.8)/Post: 13.0 (6.1)/*p-*value = 0.0712 Hip angle at contact, adduction-abduction: Pre: 180.5 (6.0)/Post: 189.9 (7.7)/*p-*value = 0.000 * Hip angle at toe off, adduction-abduction: Pre: 182.1 (8.0)/Post: 187.3 (10.7)/*p-*value = 0.071 - Passive ROM, mean (SD): Hip flexion (knee extended): Pre: 93.8 (11.4)/Post: 100.3 (7.0)/*p-*value = 0.034 * Hip flexion (knee flexed): Pre: 128.8 (9.1)/Post: 126.0 (10.6)/*p-*value = 0.154 Hip extension: Pre: 16.1 (3.8)/Post: 14.0 (2.5)/*p-*value = 0.092 Hip abduction: Pre: 32.6 (9.2)/Post: 40.0 (10.8)/*p-*value = 0.0006 * Hip adduction: Pre: 24.6 (9.7)/Post: 37.0 (13.0)/*p-*value = 0.001 * Hip external rotation: Pre: 31.0 (5.6)/Post: 40.6 (6.9)/*p-*value = 0.000 * Hip internal rotation: Pre: 34.3 (7.9)/Post: 37.8 (7.2)/*p-*value = 0.109 Knee flexion: Pre: 139.4 (6.4)/Post: 140.6 (4.7)/*p-*value = 0.125 Ankle plantar-flexion: Pre: 48.8 (6.0)/Post: 47.6 (3.6)/*p-*value = 0.503 Ankle dorsi-flexion: Pre: 10.2 (3.3)/Post: 11.5 (3.8)/*p-*value = 0.155 Subtalar inversion: Pre: 11.0 (2.3)/Post: 11.7 (2.6)/*p-*value = 0.263 Subtalar eversion: Pre: 8.2 (3.2)/Post: 9.3 (1.9)/*p-*value = 0.227
Husted, 1999 [24] (Non-randomized, non-controlled)	*n* = 19 (84.2% women) Score EDSS: NR Age: NR Disease duration: NR MS type: RR: *n* = 11 (58%) PP: *n* = 5 (26%) NR: *n* = 4 (16%)	“Tai Chi program” Duration: 8 weeks Development: Volume: 1 h Frequency: 2 times/week	Flexibility: - Posterior kinetic chain (Hamstring flexibility test)	- Hamstring flexibility test: Pre: −5.3 cm/Post: −3.8 cm Change: 28%

IG: Intervention group; CG: Control group; EDSS: Expanded Disability Status Scale; NR: Not reported; MS: Multiple Sclerosis; RR: Relapsing-Remitting; SP: Secondary Progressive; PP: Primary Progressive; RPE: Rating of Perceived Exertion; Post-adjusted: Adjusted for sex, age, baseline value; ROM: Range of motion; ThSp1: First thoracic vertebrae; S1: First sacral vertebrae. *: Significant values *p*-value ≤ 0.05.

**Table 2:** Physiotherapy Evidence Database (PEDro) results of the methodological quality evaluation of the randomized controlled trials.

	First Author, Year			
Criteria (1–11)	McAuley, 2015 [18]	Duff, 2018 [19]	Pau, 2017 [20]	Ponzano, 2017 [21]
**1. Eligibility criteria**	YES *	YES *	YES *	YES *
**2. Random allocation**	YES	YES	YES	YES
**3. Concealed allocation**	YES	NO	YES	NO
**4. Baseline comparability**	YES	YES	YES	NO
**5. Blind subjects**	NO	NO	NO	NO
**6. Blind therapists**	YES	NO	NO	NO
**7. Blind assessors**	YES	YES	NO	NO
**8. Key outcome (+85% subjects)**	YES	YES	YES	YES
**9. Intention-to-treat-analysis**	YES	YES	YES	NO
**10. Between-group comparisons**	YES	YES	YES	NO
**11. Point estimates and variability**	YES	YES	YES	YES
**Score (0–10)**	9/10	7/10	7/10	3/10

* Not included in total score.

**Table 3:** Results of the methodological quality evaluation of the non-randomized controlled trials.

	First Author, Year		
Criteria (1–12)	Pereira, 2012 [22]	Rodger, 1999 [23]	Husted, 1999 [24]
**1. Was the study question or objective clearly stated?**	YES	YES	NO
**2. Were eligibility/selection criteria for the study population prespecified and clearly described?**	YES	NO	NO
**3. Were the participants in the study representative of those who would be eligible for the test/service/intervention in the general or clinical population of interest?**	CD	CD	CD
**4. Were all the eligible participants that met the prespecified entry criteria enrolled?**	YES	YES	YES
**5. Was the sample size sufficiently large to provide confidence in the findings?**	NO	NO	NO
**6. Was the test/service/intervention clearly described and delivered consistently across the study population?**	YES	YES	NO
**7. Were the outcome measures prespecified, clearly defined, valid, reliable, and assessed consistently across all study participants?**	YES	YES	YES
**8. Were the people assessing the outcomes blinded to the participants’ exposures/interventions?**	NO	NO	NO
**9. Was the loss to follow-up after baseline 20% or less? Were those lost to follow-up accounted for in the analysis?**	NO	NO	NO
**10. Did the statistical methods examine changes in outcome measures from before to after the intervention? Were statistical tests done that provided *p***-**values for the pre-to-post changes?**	YES	YES	NO
**11. Were outcome measures of interest taken multiple times before the intervention and multiple times after the intervention (i.e., did they use and interrupted time-series design)?**	YES	NO	NO
**12. If the intervention was conducted at a group level (e.g., a whole hospital, a community, etc.) did the statistical analysis take into account the use of individual-level data to determine effects at the group level?**	NO	NO	NO
**Score (0–12)**	7/12	5/12	2/12

CD: Cannot determine; NA: Not applicable; NR: Not reported.

**Table 4:** Results of the methodological quality evaluation of the scale Consensus on Exercise Reporting Template.

Criteria (1–16)	First Author, Year						
	McAuley, 2015 [18]	Duff, 2018 [19]	Pau, 2017 [20]	Ponzano, 2017 [21]	Pereira, 2012 [22]	Rodgers, 1999 [23]	Husted, 1999 [24]
**1. Description of the type of exercise equipment**	YES	YES	YES	YES	YES	YES	NO
**2. Description of the qualifications, expertise and/or training**	YES	YES	YES	NO	NO	NO	NO
**3. Describe: exercises are individual or in a group**	YES	YES	YES	NO	YES	YES	YES
**4. Describe: exercises are supervised or unsupervised; how they are delivered**	YES	YES	YES	NO	YES	NO	YES
**5. Detailed description of how adherence to exercise is measured and reported**	YES	NO	NO	NO	YES	NO	NO
**6. Detailed description of motivation strategies**	YES	NO	NO	NO	NO	NO	NO
**7a. Detailed description of the decision rule(s) for determining exercise progression**	YES	YES	YES	NO	YES	NO	NO
**7b. Detailed description of how the exercise program was progressed**	YES	NO	YES	NO	YES	NO	NO
**8. Detailed description of each exercise to enable replication**	YES	NO	YES	YES	YES	NO	NO
**9. Detailed description of any home program component**	YES	NO	YES	YES	NO	NO	NO
**10. Describe whether there are any non-exercise components**	YES	YES	NO	NO	NO	NO	NO
**11. Describe the type and number of adverse events that occur during exercise**	NO	YES	YES	NO	YES	NO	NO
**12. Describe the setting in which the exercises are performed**	YES	YES	YES	NO	YES	YES	YES
**13. Detailed description of the exercise intervention**	YES	YES	YES	YES	YES	YES	YES
**14a. Describe whether the exercises are generic (one size fits all) or tailored**	YES	YES	YES	YES	YES	YES	YES
**14b. Detailed description of how exercises are tailored to the individual**	YES	NO	YES	NO	YES	YES	NO
**15. Describe the decision rule for determining the starting level**	NO	NO	YES	YES	NO	NO	NO
**16a. Describe how adherence or fidelity is assessed/measured**	YES	NO	YES	NO	NO	NO	NO
**16b. Describe the extent to which the intervention was delivered as planned**	YES	YES	YES	YES	YES	YES	YES
**Score (0–19)**	17/19	11/19	16/19	7/19	13/19	7/19	6/19

**Table 5:** Effect size (Cohen’s *d*) of the interventions.

First Author, Year	Variable	Group Comparison	Cohen’s *d*	95% CI	
				Lower Limit	Upper Limit
McAuley, 2015 [18]	Sit and reach test	IG pre vs post	0.19	−0.38	0.75
		CG pre vs post	−0.06	−0.62	0.51
		IG vs CG	−0.25	−0.82	0.31
	Back Scratch test	IG pre vs post	−0.27	−0.84	0.30
		CG pre vs post	0.85	0.26	1.44
		IG vs CG	1.12	0.51	1.73
Duff, 2018 [19]	Sit and Reach test	IG pre vs post	0.17	−0.54	0.89
		CG pre vs post	0.19	−0.53	0.90
		IG vs CG	−0.01	−0.73	0.71
Pau, 2017 [20]	ROM gait Hip flexion–extension	IG pre vs post	0.42	−0.42	1.27
		CG pre vs post	0.16	−0.67	1.00
		IG vs CG	0.44	−0.40	1.29
	ROM gait Knee flexion–extension	IG pre vs post	0.47	−0.38	1.32
		CG pre vs post	0.08	−0.75	0.92
		IG vs CG	0.31	−0.54	1.15
	ROM gait Ankle dorsi–plantar–flexion	IG pre vs post	0.39	−0.46	1.23
		CG pre vs post	0.01	−0.82	0.85
		IG vs CG	0.30	−0.54	1.14
Rodgers, 1999 [23]	ROM gait Ankle dorsi-flexion	IG pre vs post	0.84	0.16	1.53
	ROM gait Ankle plantar-flexion	IG pre vs post	0.91	0.22	1.60
	ROM gait Ankle (dorsi-plantar-flexion)	IG pre vs post	0.26	−0.40	0.91
	ROM gait Ankle angle at contact	IG pre vs post	1.20	0.48	1.91
	ROM gait Ankle angle at toe-off	IG pre vs post	0.65	−0.02	1.32
	ROM gait Knee flexion	IG pre vs post	0.17	−0.48	0.83
	ROM gait Knee extension	IG pre vs post	−0.14	−0.80	0.51
	ROM gait Knee (flexo-extension)	IG pre vs post	−0.26	−0.92	0.39
	ROM gait Knee angle at contact	IG pre vs post	0.06	−0.59	0.72
	ROM gait Knee angle at toe off	IG pre vs post	0.10	−0.56	0.75
	ROM gait Hip extension	IG pre vs post	−0.59	−1.26	0.08
	ROM gait Hip flexion	IG pre vs post	−0.01	−0.67	0.64
	ROM gait Hip (flexo-extension)	IG pre vs post	−0.72	−1.40	−0.04
	ROM gait Hip angle at contact (flexo-extension)	IG pre vs post	0.13	−0.52	0.79
	ROM gait Hip angle at toe off (flexo-extension)	IG pre vs post	−0.44	−1.10	0.22
	ROM gait Hip adduction	IG pre vs post	1.24	0.52	1.96
	ROM gait Hip abduction	IG pre vs post	2.06	1.23	2.88
	ROM gait Hip (adduction–abduction)	IG pre vs post	−0.53	−1.20	0.14
	ROM gait Hip angle at contact (adduc–abduc)	IG pre vs post	1.33	0.60	2.06
	ROM gait Hip angle at toe off (adduction–abduction)	IG pre vs post	0.54	0.13	1.21
	PROM Hip flexion (knee extended)	IG pre vs post	0.67	0.00	1.35
	PROM Hip flexion (knee flexed)	IG pre vs post	−0.28	−0.93	0.38
	PROM Hip extension	IG pre vs post	−0.64	−1.31	0.03
	PROM Hip abduction	IG pre vs post	0.72	0.04	1.40
	PROM Hip adduction	IG pre vs post	1.06	0.35	1.76
	PROM Hip external rotation	IG pre vs post	1.49	0.75	2.24
	PROM Hip internal rotation	IG pre vs post	0.45	−0.21	1.12
	PROM Knee flexion	IG pre vs post	0.21	−0.45	0.86
	PROM Ankle plantar-flexion	IG pre vs post	−0.24	−0.89	0.42
	PROM Ankle dorsi-flexion	IG pre vs post	0.36	−0.30	1.02
	PROM Subtalar inversion	IG pre vs post	0.28	−0.38	0.94
	PROM Subtalar eversion	IG pre vs post	0.41	−0.25	1.07

CG: Control Group; CI: Confidence Interval; IG: Intervention Group; ROM: Range of Motion; PROM: Passive Range of Motion.
